# JCD Editorial October 2024

**DOI:** 10.1111/jocd.16594

**Published:** 2024-09-26

**Authors:** Michael H. Gold

**Affiliations:** ^1^ Gold Skin Care Center Tennessee Clinical Research Center Nashville Tennessee USA

Welcome to the October 2024 issue of the Journal of Cosmetic Dermatology. It is hard for me to imagine that we are winding down 2024. It feels that time is moving very fast these days and for some that may be a good thing while others want to see time slow down, even for a little while, to catch one's breath or to relax with family and friends, or to do nothing for a period of time. For many of us that is not a luxury that we have, so it remains full steam ahead for most of us, and that is not a bad thing either.

JCD remains your preferred journal; we want to be your source for scientific clinical work and writings encompassing the gamut of cosmetic and aesthetic dermatology. As we work our way into October, we are happy to report that we are receiving more and more article submissions, and that these articles are of the highest quality in the aesthetic space. Keep them coming to us and we will do everything we can to move them through our rigorous peer‐review process. We also will keep doing our best to publish your articles in a timely fashion—and not hold it for upwards of a year or more before seeing a published manuscript. We want to do things efficiently and correctly to assure that articles receive the care that they are entitled to and we promise everyone that we do this every day.

Welcome to JCD and we hope you enjoy these amazing articles.

